# Prevalence, patterns, and predictors of diarrhea: a spatial-temporal comprehensive evaluation in India

**DOI:** 10.1186/s12889-018-6213-z

**Published:** 2018-11-23

**Authors:** Nilima, Asha Kamath, Karthik Shetty, B. Unnikrishnan, Siddharth Kaushik, Shesh N. Rai

**Affiliations:** 1 Department of Statistics, Prasanna School of Public Health, Manipal Academy of Higher Education, Karnataka, Manipal, 576104 India; 2 Department of Community Medicine, Manipal Academy of Higher Education, Karnataka, Manipal, 576104 India; 3 V-4 division, Central Scientific Instruments Organization, Chandigarh, India; 4 Biostatistics Shared Facility, James Graham Brown Cancer Center, University of Louisville, Louisville, KY USA; 5 Department of Bioinformatics & Biostatistics, School of Public Health & Information Sciences, University of Louisville, Louisville, KY USA

**Keywords:** Hotspot, Spatial regression, Spatial lag-model, Spatial error model, India

## Abstract

**Background:**

Spatial analysis has been vital in mapping the spread of diseases and assisting in policy making. Targeting diarrhea transmission hotspots is one of the potential strategies for reducing diarrhea cases. This study aimed to examine the spatial-temporal variations and to identify the modifiable determinants of diarrhea while controlling for the spatial dependence in the data.

**Methods:**

An ecological study on diarrhea data from DLHS-3 and NFHS- 4 in India. Moran’s I and LISA were used to detect the spatial clustering of diarrhea cases and to test for clustering in the data. Spatial regression was used to identify the modifiable factors associated with the prevalence of diarrhea. The study comprised of the prevalence of diarrhea among the children below the age of five years (U-5 s) across different states in India. The determinants of diarrhea were obtained using spatial lag models. The software used were GeoDa 1.6.6 and QGIS 2.0.

**Results:**

The presence of spatial autocorrelation in DLHS-3 and NFHS-4 (Moron’s *I* = 0.577 and 0.369 respectively) enforces the usage of geographical properties while modeling the diarrhea data. The geographic clustering of high-prevalence districts was observed in the state of UP consistently. The spatial pattern of the percentage of children with diarrhea was persistently associated with the household with a sanitation facility (%) (*p* = 0.023 and *p* = 0.011). Compared to the diarrhea cases in the period 2007–2008, no much reduction was observed in the period 2015–2016. The prevalence of diarrhea and percentage of household with sanitation were ranging between 0.1–33.8% and 1.3–96.1% in the period 2007–2008 and 0.6–29.1% and 10.4–92.0% in the period 2015–2016 respectively. The least and highest prevalence of diarrhea being consistently from Assam and UP respectively.

**Conclusion:**

Despite improvements in controlling spread of diarrheal disease, the burden remains high. Focus on widespread diarrheal disease control strategy by addressing the social determinants of health like basic sanitation is crucial to reduce the burden of diarrhea among U-5 s in India. The identification of hotspots will aid in the planning of control strategies for goal setting in the targeted regions.

**Electronic supplementary material:**

The online version of this article (10.1186/s12889-018-6213-z) contains supplementary material, which is available to authorized users.

## Background

The time between birth and age 5 of a child’s life represents a sensitive period for development [1, 2]. The developmental construct of the physical health and well-being of a U-5 includes p*hysical readiness for the school day, physical liberation, and gross and fine motor skills.* Diarrhea is a gastrointestinal infection caused by pathogenic microorganisms including *E. coli*, Rotavirus, Salmonella spp., Vibrio cholerae, Campylobacter, and Shigella [3]. Highly preventable morbidity, diarrhea, is a leading cause of malnutrition and the third leading cause of mortality among U-5’s [4]. Diarrheal illness remained a key threat to global U-5 health and was estimated to be a reason for 15% of all-cause mortality among U-5 s [5]. Globally 1.339 billion U-5 s and 79 thousand neonates aged 0–27 days were estimated to be dead due to diarrhea [6]. India, Nigeria, Afghanistan, Pakistan, and Ethiopia accounts for more than half of the deaths caused by diarrhea [7, 8].

To prevent diarrheal disease and to monitor and control U-5MR, the government of India introduced schemes including child survival and safe motherhood programme in 1992, target-free approach in 1996, reproductive and child health programme-1 in 1997, reproductive and child health programme-2 in 2005, national rural/urban health mission between 2005 and 2012, national health mission since 2013 to-date, the expanded program on immunization, and a program for the control of diarrheal diseases and acute respiratory infection. As a result of various efforts, India has recorded a decline of 52.73% in U-5MR with 55 and 29 deaths per 1000 live births in 2011 and 2015 respectively [9–12]. India has set its way to achieve the SDG target for under-five child mortality of 25 by 2030 [13, 14]. The average annual rate of decline in U-5MR at 3.1% in India was considered insufficient with the MDG- 4 target to minimize the U-5 mortality to 29/1000 live births by 2015 [15–17]. To prevent U-5MR and to achieve the various targets, special attention need to be put on most prevalent diseases among U-5 s including diarrhea. Prevalence of diarrhea is highly heterogeneous across the states of India, and it ranges from 0.1–33.8% in period 2007–2008 and 0.6–29.1% in period 2015–2016 [18, 19]. Though the reduction in the range of diarrhea prevalence over time is observed, generating more evidence through spatial analysis will help target the ones at the rear. Spatial analysis is shown to be important in mapping the extent of infectious diseases and assisting the control policies [20–23]. *S*tudies examined cultural dimensions of neighborhoods, conveying critical factors that add to the ethnic divisions among spaces in the unequal distribution of developmental health [1]. The spatial techniques help us identify the hotspots and provide the information on significant clusters and the associated factors. The spatial evaluation will enable public health officers and policymakers for strategic planning to reduce the prevalence of diarrhea. More than 1 billion people worldwide are deprived of basic facilities including clean water sources and sanitation [20]. In light of recent developments in India, evidence on diarrheal diseases and its determinants need to be studied for improved planning and need-based organization of health services within the country. Targeting the hotspots may represent an efficacious strategy for further reduction in diarrhea cases. Developing countries like India, with highly heterogeneous diarrhea occurrence, may benefit the most from a targeted community-wide interventional approach that reaches all diarrhea-affected and diarrhea-exposed children. On a priority basis, interventions should be channeled to the most affected districts in India, as revealed by the hotspot analysis. Availability of effective interventions to the locations at the rear can prevent diarrheal occurrence [24].

To the best of our knowledge, the spatial-temporal analysis of diarrhea prevalence in India has not been previously reported. In the present study, the spatiotemporal mappings of regions affected by the pervasive illnesses are reported for instituting the relation between the disease and the dynamics associated with its evolution. The mapping will assist in the formulation and implementation of need-based preventive strategies for the diarrheal disease. This study aims at addressing the social determinants of health to reduce the burden of diarrhea among U-5 s in India.

## Methods

### Dataset

**Fig. 1:**
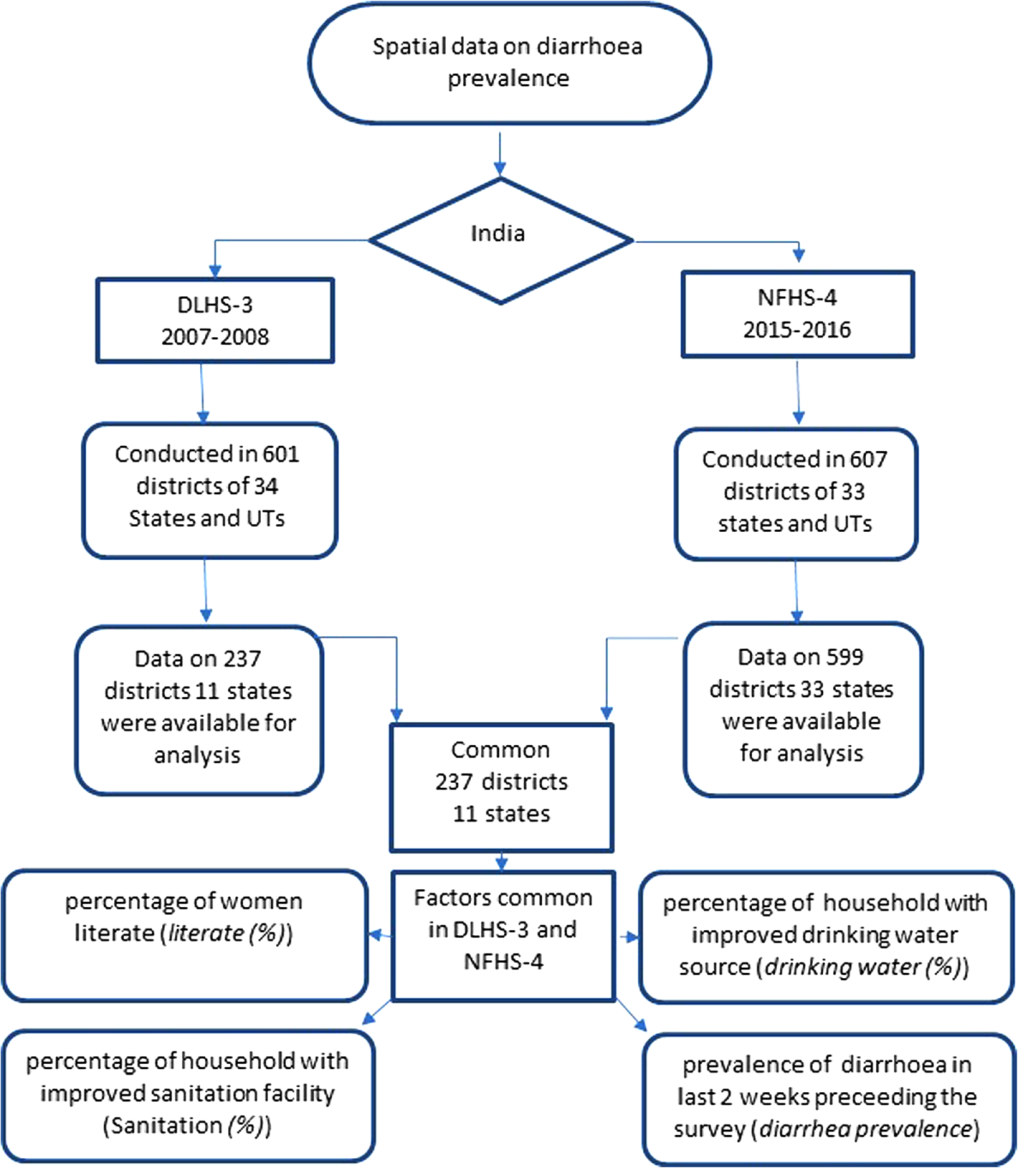
Schematic presentation of the data collected by the national agency and the data used for the analysis in the present study

This ecological study uses the aggregate level data from a large-scale multi-round survey conducted in a representative sample of households in India. The surveys were conducted by various regional agencies and coordinated by the IIPS, Mumbai. In the present study, we use the third round of the DLHS-3 [18] and the fourth round of the NFHS-4 [19] conducted in the period 2007–2008 and 2015–2016 respectively. Data was obtained (from http://rchiips.org) under the National Data Sharing and Accessibility Policy (NDSAP) of Government of India and is freely available and accessible. The data on EAG states, Daman and Diu, Dadra and Nagar Haveli and Lakshadweep, were not available at both the time points and hence are not analyzed. The schematic presentation of data used in the analysis is shown in Fig. 1.

In addition to the common variables data, DLHS-3 provides data on the percentage of the pucca house [*pucca house(%)*], women aware about diarrhea *[awareness (%)]* and households with BPL card [*BPL (%)*] [18]. The present study utilizes data on the above-listed variables for the analysis. The software used in this study was GeoDa [25] 1.6.6 (Arizona State University, Tempe, AZ, USA) and QGIS 2.0. [26]. The outcome variable considered in this study was the district level prevalence of diarrhea.

### Exploratory spatial analysis

Spatial cluster detection was performed to identify the cluster of locations with a higher prevalence of diarrhea for the formulation of policies on MCH. Global spatial autocorrelation Moran’s I was used to measure the overall clustering of the data and to project the strength and pattern of spatial autocorrelation. LISA statistic was used to detect the hot spots of the district-level percentage of children suffering from diarrhea [27]. LISA was used to assess the influence of individual districts on the magnitude of the global statistic and to identify the clusters [28]. A significance map shows the areas with a statistically significant LISA statistic value [29]. Hotspots are the geographical units with *high* diarrhea prevalence and are surrounded by other geographic units with *high* diarrhea prevalence. Similarly, a cold spot is a geographic location with *low* diarrhea prevalence surrounded by other geographic units with *low* diarrhea prevalence. *High-high* and *low-low* districts suggest the clustering of geographies with similar values of diarrhea prevalence whereas the *high-low* and *low*-*high* districts indicate spatial outliers [30]. Queens first order contiguity matrix was used to provide the weights [31]. A *p* < 0.05 was considered significant throughout.

### Confirmatory spatial analysis

To explore the relationship between the percentage of diarrhea and a set of explanatory variables regression techniques were used. The application of appropriate regression technique depends on the violation of assumptions required. The OLS estimation is built on assumptions that random error terms or independent variables are uncorrelated, have a constant variance and follow a normal distribution. The estimates obtained using OLS are biased and inefficient when the above assumptions are violated [32]. Multicollinearity condition number was used to investigate if the independent variables are correlated. The Breusch-Pagan test was used to test for heteroskedasticity. Normality of error distribution was assessed using the Jarque-Bera test of normality of regression disturbances. Spatial regression is a technique used to predict the value of an outcome variable based on values of a set of explanatory variables, taking into account the spatial dependence. Two forms of spatial dependence are a spatial error and spatial lag. The dependency is said to be on the spatial error if the error terms across different spatial units are correlated. The spatial dependence is accounted to spatial lag if the dependent variable y in place i is affected by the independent variables in both places i and j [33–36]. The spatial error model with the vector of spatially autocorrelated error term (ε), the matrix of weights indicating the spatial framework of neighborhood effects among the residuals (*W*), the spatial error coefficient (λ), the vector of spatially autocorrelated error term (u), and vector of uncorrelated error terms (ε), is mentioned in eq. (1).

1 $$ \mathrm{y}\kern0.5em =\kern0.5em \mathrm{X}\upbeta +\mathrm{u}\kern5em \mathrm{where}\kern0.5em \mathrm{u}\kern0.5em =\kern0.5em \uplambda \mathrm{Wu}+\kern0.5em \upvarepsilon $$

If the spatial error coefficient λ = 0, then it is said to exist no spatial correlation between the errors. The spatial lag model for the matrix of weights indicating the spatial framework of neighborhood effects among the dependent variable (*W*), the matrix of observations on the explanatory variable (X), the vector of error terms (ε), and the parameters β and *ρ*, is mentioned in eq. (2).

2 $$ \mathrm{y}\kern0.5em =\kern0.5em \mathrm{XB}\kern0.5em +\kern0.5em \uprho \mathrm{Wy}\kern0.5em +\kern0.5em \upvarepsilon $$

**Fig. 2:**
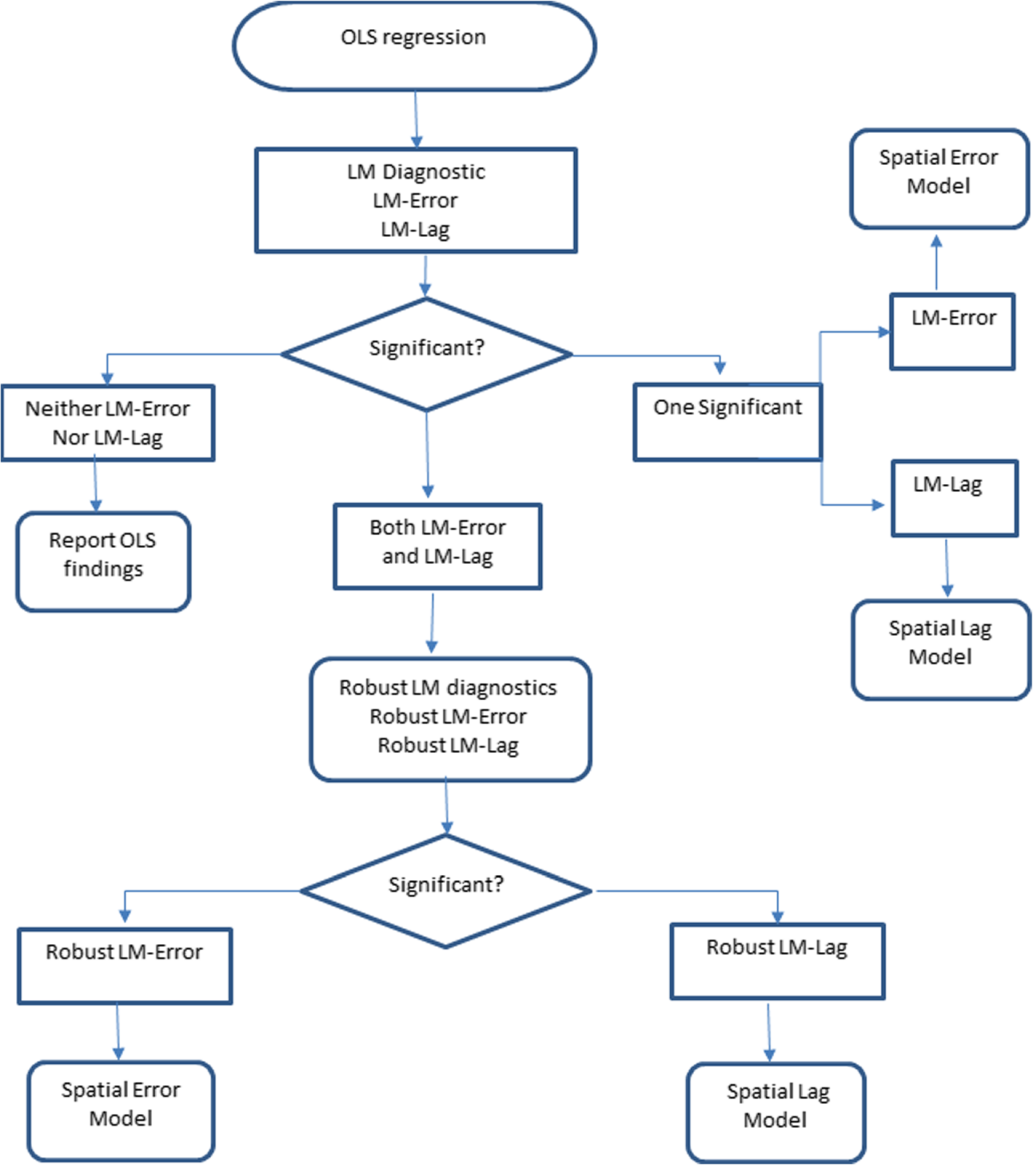
Schematic for the choice of the most suitable model among OLS, spatial lag & spatial error models

A spatial lag is a variable that averages the neighboring values of a location and controls spatial autocorrelation in the dependent variable. Robust LM(error) and Robust LM(lag) test were used to investigate the spatial dependence. The analysis mentioned above were performed using GeoDA software. The schematic for the choice of an appropriate model for the data is depicted in Fig. 2.

Bivariate LISA will be then used to examine the spatial relationship between the exposure and the response variable across the geographic locations of India.

## Results

### Overall clustering of data

**Fig. 3:**
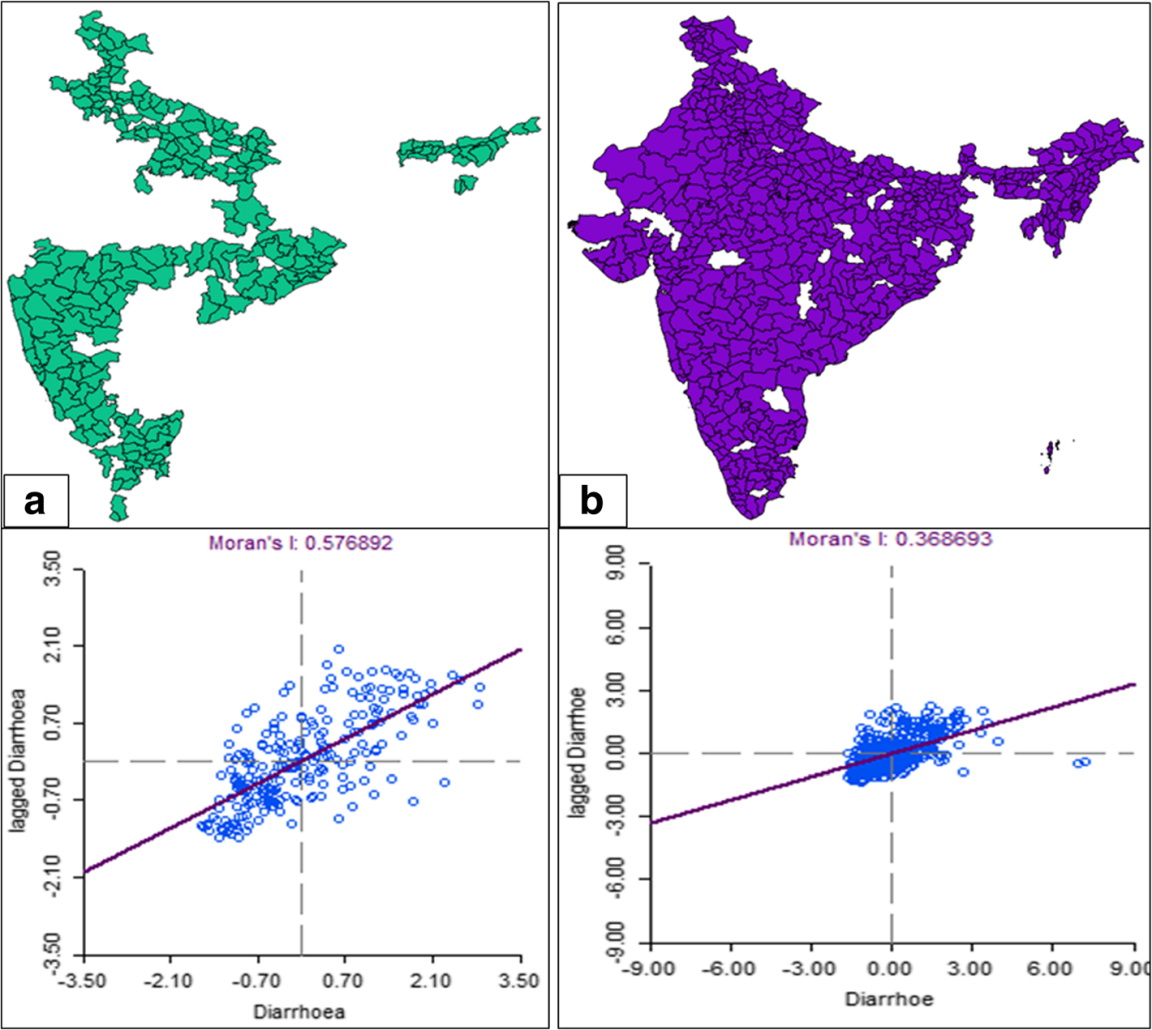
The shapefile of India and the Moran’s scatter plot for diarrhea prevalence in India in the period **(a)** 2007–2008 **(b)** 2015–2016

Moron’s scatter plot in Fig. 3 provides a visual representation of data attached to the shapefile and the spatial associations in the neighborhood of each district-level observation. The Global spatial autocorrelation Moran’s I value for the prevalence of diarrhea is observed to decrease significantly over time from 0.579 to 0.369 when investigated using the data from DLHS-3 and NFHS-4 respectively. The result indicates segregation of elements of the significant clusters over time and is suggestive of the need to include a component of geography in the model for identifying the determinants of diarrhea.

### Descriptives

Table 1 highlights that the least and the most diarrhea prevalent districts were consistently from the states of Assam and UP, respectively.

**Table 1:** The prevalence of diarrhea and the most endemic and non-endemic states in India

Diarrhea*				
Period	Average Prevalence	95% Confidence Interval	Least prevalent district, state, prevalence	Most prevalent district, state, prevalence
2015–2016	0.088	(0.0818, 0.09331)	Udalguri, Assam, 0.006	Siddharthnagar, UP, 0.291
2007–2008	0.121	(0.1111, 0.1303)	Nagaon, Assam, 0.001	Kheri, UP, 0.338

*Reported only for the common 11 states in the period 2007–2008 and 2015–2016

### Hotspot detection

**Fig. 4:**
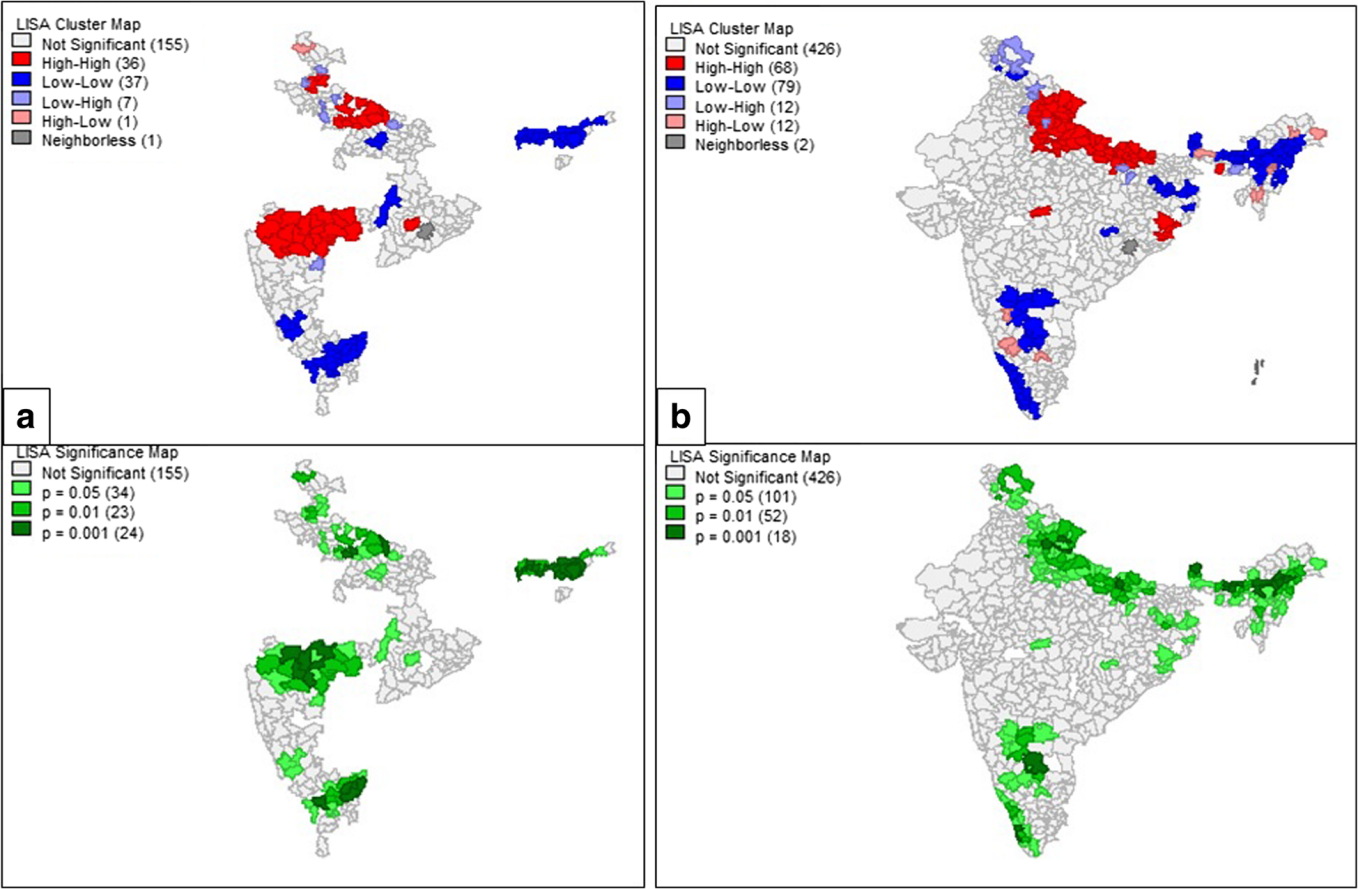
Local indicators of spatial association cluster map and the significance map for diarrhea prevalence in the study region in the year (**a**) 2007–2008 (**b**) 2015–2016

This study revealed the presence of 32 hotspots and 63 coldspots of diarrhea in the study region using the DLHS 3 data. The hotspots were observed to exist in the districts of Haryana, North-west UP, North and East Maharashtra, and North-West Orissa. Coldspots existed in the districts of East Karnataka, North Tamil Nadu, Chhattisgarh, and Assam. NFHS4 hotspots were observed in the Uttarakhand, UP, Madhya Pradesh, Odisha, and Meghalaya. Coldspots were observed in the states of Assam, Andhra Pradesh, Sikkim, and Kerala. The significance map showed locations with significant local Moran statistics as depicted in Fig. 4. The observed blank places on the map of India are due to the unavailability of data for empowered action group states. The list of spatial outliers and the statistically significant clusters which contributed to the global autocorrelation measure are listed in Additional file 1: Tables S1 and S2.

### Spatial regression

On the data derived from DLHS-3 and NFHS-4 respectively, the diagnostic tests for OLS assumptions suggest avoidable dependencies between the explanatory variable with the multicollinearity condition number 20.403 and 22.624. A violation of assumption on normally distributed errors surveys using the Jarque-Bera test was observed (*p* < 0.001 & p < 0.001). The Breusch-pagan test indicated the possible existence of heteroskedasticity (*p* < 0.001 & < 0.001), due to the spatial dependence in the data, justifying the use of spatial regression. The estimates obtained using various models are shown in Table 2.

**Table 2:** Estimated regression coefficients obtained from OLS, spatial lag and spatial error models

Year	Variables	OLS model			Spatial Lag model			Spatial Error model		
		Coeff.	SE	*p*-value	Coeff.	SE	*p*-value	Coeff.	SE	*p*-value
2015–2016	Constant	6.301	1.767	< 0.001	4.149	1.621	0.010	10.136	2.065	< 0.001*
	Sanitation	− 0.053	0.014	< 0.001*	−0.030	0.012	0.011*	− 0.039	0.016	0.016*
	Lambda	–	–	–	–	–	–	0.498	0.045	< 0.001
	Rho	–	–	–	0.482	0.045	< 0.001*	–	–	–
2007–2008	Constant	13.396	2.485	< 0.001*	4.671	2.093	0.025*	11.285	3.007	< 0.001*
	Awareness	−0.146	0.027	< 0.001*	−0.089	0.021	< 0.001*	− 0.097	0.026	< 0.001*
	Illiterate	0.078	0.050	0.114	0.077	0.039	0.046*	0.110	0.049	0.025*
	Water Supply	−0.058	0.016	< 0.001*	−0.036	0.013	0.005*	− 0.050	0.021	0.015*
	Sanitation	−0.081	0.027	0.003*	−0.048	0.021	0.023*	− 0.067	0.031	0.029*
	PuccaHouse	0.153	0.039	< 0.001*	0.072	0.031	0.021*	0.101	0.046	0.028*
	BPL	0.066	0.033	0.046*	0.039	0.026	0.134	0.013	0.054	< 0.001*
	Lambda	–	–	–	–	–	–	0.604	0.054	< 0.001*
	Rho	–	–	–	0.569	0.055	< 0.001*	–	–	–

*p < 0.05 indicates that the factor has a significant impact on diarrhea prevalence. A factor significant in any one of the model is reported

For the model from DLHS-3**,** it was observed that the spatial error model compared to the spatial lag model had Akaike Information Criterion (1489.62 vs. 1488.24) Schwarz criterion (1513.8 vs. 1515.88). The Robust LM(error) statistic was not significant (*p* = 0.465) whereas the Robust LM(lag) statistic was significant (*p* = 0.004). The spatial lag model was observed to have the best fit for the data derived from the DLHS-3 survey. For a unit increase in the awareness on diarrhea among women (%), improved water supply (%) and sanitation facility (%), the prevalence of diarrhea is expected to decrease by 0.089, 0.036 and 0.048% respectively. With a unit increase in illiterate (%) and pucca house, we expect 0.077 and 0.072% increase in the diarrhea prevalence. On the same lines, for the model obtained based on data derived from NFHS-4**,** it was observed that spatial error model compared to the spatial lag model, had Akaike Information Criterion (3507.93 vs. 3507.15) and Schwarz criterion (3525.52 vs 3529.13). The Robust LM (error) statistic was not significant (*p* = 0.764) whereas the Robust LM (lag) statistic was significant (*p* = 0.003). The spatial lag model was observed to have the best fit and is hence used for further discussion. It is to be noted that after a significant eight years gap, improved sanitation facility was observed to have a persistent significant association with the diarrhea prevalence in India. It was observed that with a unit increase in the improved sanitation facility (%), the diarrhea prevalence is expected to decrease by 0.031%.

Table 3 highlights that the districts with the lowest and the highest percentage of household with proper sanitation.

**Table 3:** The descriptive for sanitation facility in India over the specified period

Sanitation*				
Period	Average Percentage	95% Confidence Interval	Lowest percentage district, state, percentage	Highest percentage district, state, percentage
2015–2016	47.97	(45.597, 50.336)	Shrawasti, UP, 10.4	Dakshina Kannada, Karnataka, 92.0
2007–2008	30.31	(27.257, 33.372)	Malkangiri, Odisha, 1.3	Hailakandi, Assam, 96.1

*Reported only for the common 11 states in the period 2007–2008 and 2015–2016

**Fig. 5:**
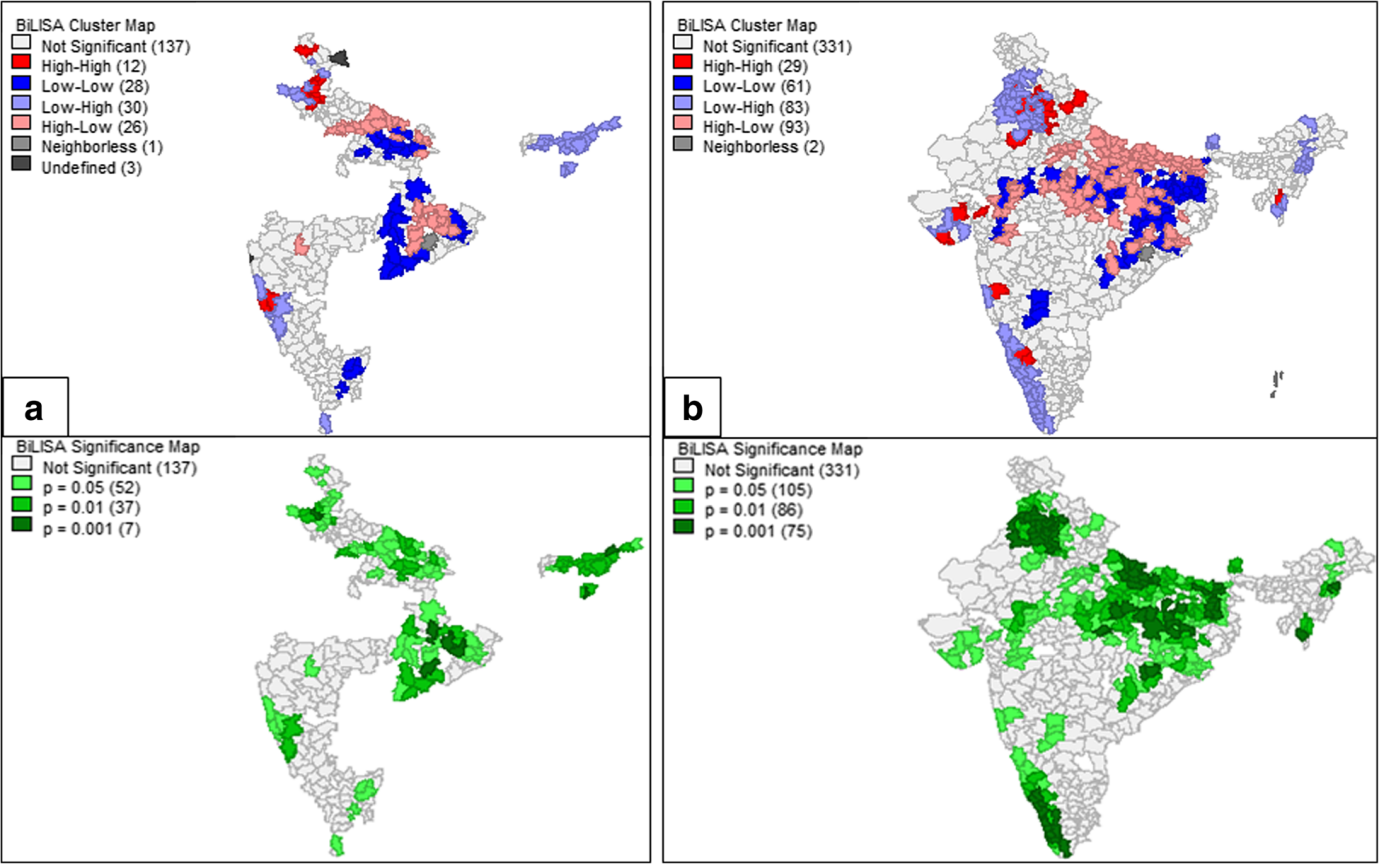
Bivariate Local indicators of spatial association cluster map and the significance map for the prevalence of diarrhea with the sanitation (%) in the study region in the year (**a**) 2007–2008 (**b**) 2015–2016

Bivariate LISA is used to address if the geographic locations underprivileged in terms of sanitation facility were also lacking in terms of diarrhea prevention. The Bivariate LISA map shows the local patterns of spatial correlation between the diarrhea prevalence of a district and the average sanitation facility for its neighbors. The results are presented in Fig. 5.

## Discussion

The present study findings provide valuable insights on factors affecting diarrhea prevalence. The spatial evaluation of evidence on diarrhea conceals the fact that the situation has improved with time. Most of the states have shown good progress in reducing the prevalence of diarrhea among U-5 except UP and Odisha which remained persistent hotspot till 2015–2016. The state of Maharashtra, Punjab, and Haryana had several hotspots in period 2007–2008 which were no more significant in the period 2015–2016. The results signify that the strategic approach of the government is fruitful and the sweats of focusing on low performing states are paying off. These remarkable achievements are the result of various initiatives of the Government [14]. The implemented community intervention National Rural Health Mission between 2005 and 2012 might have assisted in reducing the spatial inequalities in diarrhea indicators. The observed reduction may also be a result of the state government schemes like Punjab rural water supply and sanitation project [37]. The solid waste disposal concessions awarded to 50 municipal corporations in India including Kolhapur district of Maharashtra would have contributed to decreasing diarrheal disease prevalence in the state of Maharashtra [38]. The impact on improved water and sanitation could be a fruit of several other national and international projects including the second phase of community-led infrastructure finance facility-2015 [39] and sulabh international [40]. Despite Nirmal Bharat Abhiyan and other schemes in the state of UP and Odisha, no significant improvement was noted. In the period 2007–2008, moderate spatial autocorrelation was noted, and the factors having significant impact on diarrhea prevalence were illiteracy and awareness among mothers, use of an improved source of drinking water, sanitation facility, living in the pucca house and having a BPL card. After eight significant years, in the period 2015–2016, a mild spatial autocorrelation was still present, and a household with improved sanitation facility (%) was observed to be solely contributing towards diarrhea prevalence. The results highlight the importance of having a better sanitation facility in every household. Studies [41, 42] with a similar finding report that the mode of water transportation, the existence of excess water in the street, domestic water storage conditions, feces disposal and the presence of vectors predispose the under-five children to diarrhea. About 2.4 billion people lacked access to proper sanitation facility, and four out of five of these underprivileged people lived in Asia alone [38, 43]. The millennium development goal to halve the proportion of people without access to basic sanitation by 2015 [38] seems unmet. To respond to these public health concerns, urban India needs to have universal access to toilets with safe collection and treatment of human excreta [44]. Indiscriminate stool disposal by the mothers, lack of hand-washing before feeding their children and hand-washing without soap need to be addressed [45, 46].

Poor sanitation not only has a direct impact on health but also has indirect contribution to the living situation, reduced education outcomes and poverty [38]. Studies [9] reveal that low socioeconomic status, poor maternal literacy, inadequate breastfeeding, malnutrition, poor sanitation and hygiene practices of the mother are associated with a higher incidence of diarrheal diseases in young children. Poor socioeconomic status is one of the factors influencing the diarrheal incidence [47]. Educational status of the mother showed a positive correlation with the incidence of diarrheal diseases [48].

The present study addressed the situation of diarrhea prevalence using the aggregate level data. The study is beneficial for region-specific allocation of resources. Despite efforts mentioned above, there are few limitations in the study. The unit of analysis is a district and not an individual. Analysing aggregate data lead to the ecological fallacy. The findings cannot be generalized at the individual level. A better identification of risk factors is expected if the meta-data with the names of finer blocks were available. Data on all the states were not available and hence were not included for the analysis. Analysis for the period 2007–2008 included data only on 237 districts compared to the period 2015–2016 data on 599 districts. This may have minor effect on the global measure of spatial autocorrelation Moran’s I, but the local measure LISA and BLISA remains unaffected. Due to the unavailability of data, factors like place of residence, wealth index, parent’s education, final say on health care, and exposure to mass media were not considered for this study. These factors might play an important role, and hence future work may utilize information on these aspect while explaining spatial dissimilarities in the prevalence of diarrhea.

## Conclusion

Despite working towards improving the modifiable risk factors, the management of diarrhea remains crucial. Proper sanitation and hygiene will help in substantial reduction of diarrhea cases. The identification of hotspots will assist in the planning of control strategies in the locations at the rear rather than plan for the whole state or nation. In addition to available schemes, customized interventions, region-specific allocation of funds and awareness programs would decrease the diarrhea prevalence, ensuring better child health.

## Additional file

Additional file 1: **Tables S1 and S2.** A list of spatial outliers and the most significant clusters are presented in Tables S1 and S2. (DOCX 20 kb)

## Abbreviations

BPL: Below Poverty line
DLHS: District Level Household and Facility Survey
EAG: Empowered action group
IIPS: International Institute of Population Sciences
LISA: Local Indicators of Spatial Association
LM: Lagrange Multiplier
MCH: Maternal and Child Health
MDG: Millennium Development Goal
NFHS: National Family Health Survey
OLS: Ordinary Least Squares
SDG: Sustainable Development Goal
U-5: Under Five
U-5MR: Under Five mortality rate
UP: Uttar Pradesh
