# Supplementary material for: Prevalence, patterns, and predictors of diarrhea: a spatial-temporal comprehensive evaluation in India
Source: BMC Public Health. 2018 Nov 23;18:1288. doi: 10.1186/s12889-018-6213-z (PMC6251155; doi:10.1186/s12889-018-6213-z)
Supplement: Supplementary file 1 — Tables S1 and S2. A list of spatial outliers and the most significant clusters are presented in Tables S1 and S2. (DOCX 20 kb) [file 12889_2018_6213_MOESM1_ESM.docx]

**SUPPLEMENTARY INFORMATION**

**Prevalence, patterns, and predictors of diarrhea: A spatial-temporal comprehensive evaluation in India**

Nilima^1^, Asha Kamath^1^, Karthik Shetty^1^, Unnikrishnan B^2^, Siddharth Kaushik^3^, and Shesh N. Rai^4,5^

^1^ Department of Statistics, Prasanna School of Public Health, Manipal Academy of Higher Education, Manipal, Karnataka- 576104, India.

^2^ Department of Community Medicine, Manipal Academy of Higher Education, Manipal, Karnataka- 576104, India

^3^ V-4 division, Central Scientific Instruments Organization, Chandigarh, India

^4^ Biostatistics Shared Facility, James Graham Brown Cancer Center

^5^ Department of Bioinformatics & Biostatistics, School of Public Health & Information Sciences, University of Louisville, Kentucky, USA

**Table S1. State-wise list of the spatial outliers of diarrhoea prevalence in India at the district level.**

| **Spatial Outlier** | | | | |
| --- | --- | --- | --- | --- |
| **Period** | **Low-High** | | **High- Low** | |
| **2007-2008** | Himachal Pradesh | Sirmaur | Himachal Pradesh | Kangra |
|  | Haryana | Kaithal | -- | -- |
|  | UP | Gonda | -- | -- |
|  | Karnataka | Bidar | -- | -- |
| **2015-2016** | Jammu and Kashmir | Reasi | Tamil Nadu | Krishnagiri |
|  | Jammu and Kashmir | Anantnag | Karnataka | Hassan |
|  | Jammu and Kashmir | Doda | Karnataka | Chikmagalur |
|  | Jammu and Kashmir | Kargil | Karnataka | Gadag |
|  | Himachal Pradesh | Sirmaur | Mizoram | Aizawl |
|  | Himachal Pradesh | Shimla | Mizoram | Mamit |
|  | Himachal Pradesh | Chamba | Arunachal Pradesh | Anjaw |
|  | UP | Ghazipur | Arunachal Pradesh | East Siang |
|  | UP | Jyotiba Phule Nagar | Nagaland | Dimapur |
|  | Bihar | Rohtas | Nagaland | Peren |
|  | Haryana | Karnal | West Bengal | Jalpaiguri |
|  | -- | -- | Meghalaya | West Garo Hills |

**Table S2. State-wise list of most significant clusters of diarrhoea prevalence in India at the district level.**

| CLUSTERS | | | | |
| --- | --- | --- | --- | --- |
|  | **HOTSPOTS** | | **COLDSPOTS** | |
| Period | **State** | **District** | **State** | **District** |
| 2007-2008 | Maharashtra | Jalan | Tamil Nadu | Erode |
|  | Maharashtra | Buldana | Assam | Bongaigaon |
|  | Maharashtra | Parbhani | Assam | Kamrup |
|  | Maharashtra | Washim | Assam | Barpeta |
|  | Maharashtra | Jalgaon | Assam | Karbi Anglong |
|  | Maharashtra | Amravati | Assam | Gola ghat |
|  | Maharashtra | Chandrapur | Assam | Nagaon |
|  | Uttar Pradesh | Farrukhabad | Assam | Marigaon |
|  | Uttar Pradesh | Bahraich | Assam | Darrang |
|  | Uttar Pradesh | Mainpuri | - | - |
|  | Odisha | Balangir | - | - |
| 2015-2016 | Uttar Pradesh | Bijnor | Assam | Sivasagar |
|  | Uttar Pradesh | Gorakhpur | Assam | Chirang |
|  | Uttarakhand | UdhamSingh Nagar | Assam | Bongaigaon |
|  | Uttarakhand | Almora | Assam | Barpeta |
|  | - | - | Assam | Baksa |
|  | - | - | Assam | Nagaon |
|  | - | - | Assam | Sonitpur |
|  | - | - | Assam | Golaghat |
|  | - | - | Assam | Lakhimpur |
|  | - | - | Assam | Jorhat |
|  | - | - | Sikkim | North |
|  | - | - | Andhra Pradesh | Anantapur |
|  | - | - | Kerala | Ernakulam |
|  | - | - | Kerala | Alappuzha |

Table S1 lists the significant outliers across different states in India. Table S2 highlights the most significant hotspots and coldspots across India. This list of hotspot will assist the researcher prioritize their efforts to the districts prone for diarrhea. Knowledge of the coldspots makes us aware of the areas performing well in the control of contagious disease like diarrhea. Spatial outliers are the unusual observation demanding attention.

******
